# Toots, tastes and tester shots: user accounts of drug sampling methods for gauging heroin potency

**DOI:** 10.1186/s12954-018-0232-z

**Published:** 2018-05-16

**Authors:** Sarah G. Mars, Jeff Ondocsin, Daniel Ciccarone

**Affiliations:** 1 Heroin in Transition Study, Department of Family and Community Medicine, UCSF, 500 Parnassus Avenue, Milberry Union East, 3rd Floor, San Francisco, CA 94143 USA; 2 Department of Family and Community Medicine, University of California, San Francisco, CA USA

**Keywords:** Overdose, Heroin, Fentanyl, Test shot, Tester shot, Injection, Insufflation, Inhalation, Harm reduction, United States

## Abstract

**Background:**

Internationally, overdose is the primary cause of death among people injecting drugs. However, since 2001, heroin-related overdose deaths in the United States (US) have risen sixfold, paralleled by a rise in the death rate attributed to synthetic opioids, particularly the fentanyls. This paper considers the adaptations some US heroin injectors are making to protect themselves from these risks.

**Methods:**

Between 2015 and 2016, a team of ethnographers collected data through semi-structured interviews and observation captured in field notes and video recording of heroin preparation/consumption. Ninety-one current heroin injectors were interviewed (Baltimore, *n* = 22; Chicago, *n* = 24; Massachusetts and New Hampshire, *n* = 36; San Francisco, *n* = 9). Experience injecting heroin ranged from < 1–47 years. Eight participants, who were exclusively heroin snorters, were also interviewed. Data were analyzed thematically.

**Results:**

Across the study sites, multiple methods of sampling “heroin” were identified, sometimes used in combination, ranging from non-injecting routes (snorting, smoking or tasting a small amount prior to injection) to injecting a partial dose and waiting. Partial injection took different forms: a “slow shot” where the user injected a portion of the solution in the syringe, keeping the needle in the injection site, and continuing or withdrawing the syringe or a “tester shot” where the solution was divided into separate injections. Other techniques included getting feedback from others using heroin of the same batch or observing those with higher tolerance injecting heroin from the same batch before judging how much to inject themselves. Although a minority of those interviewed described using these drug sampling techniques, there is clearly receptivity among some users to protecting themselves by using a variety of methods.

**Conclusions:**

The use of drug sampling as a means of preventing an overdose from injection drug use reduces the quantity absorbed at any one time allowing users to monitor drug strength and titrate their dose accordingly. Given the highly unpredictable potency of the drugs currently being sold as heroin in the US, universal precautions should be adopted more widely. Further research is needed into facilitators and barriers to the uptake of these drug sampling methods.

## Background

Heroin can be taken into the human body in a wide variety of ways, including snorting or sniffing powder or heroin solution (intranasal use), inhalation of the heated vapors (“chasing”), orally and as anal suppositories (“plugging”). Injecting, whether intravenous, subcutaneous or intramuscular, is the method of administration carrying the highest risk for multiple types of infections, overdoses and their complications [1–15]. However, despite these risks, injection appeals to users because it is the most efficient, cost-effective method of use, and intravenous injection in particular has the most intense onset of effect (“rush”). These can be important considerations when users face rising tolerance to heroin’s effects [16, 17].

Heroin “source form”—its chemical characteristics based on country of origin—also influences its mode of use [18, 19]. Bioavailability, or the measure of a drug dose that achieves circulation in the bloodstream, in turn reflects the mode of use. The three main source forms of heroin in the US—Colombian-sourced powdered heroin and both Mexican-sourced black tar heroin and powdered heroin—are all hydrochloride salts in contrast with the Afghanistan-sourced base heroin, aka “smoking heroin,” found in Europe [20]. The hydrochloride (HCL) form has relatively low bioavailability when smoked compared with base heroin [21]. Heroin HCL may be potentiated when smoked by the addition of caffeine [22], but research indicates no significant culture of heroin smoking in the US [23, 24] likely due to available source forms. Use outside of controlled conditions [25] as well as poor chasing technique [26] may also negatively affect the bioavailability of smoked heroin.

Another non-injection option to chasing/smoking is intranasal use. Intranasal heroin administration has been found to be an effective drug delivery route. It has been theorized that the lower price of US heroin in the early 1990s rather than its higher purity—as well as fears of contracting HIV—may have led to increased usage of intranasal administration [27]. US data on hospitalizations in the 1990s suggests an increase in heroin inhalation and smoking, primarily in the northeast during this period [28].

For many decades, overdose has been the primary cause of deaths among people injecting drugs [6, 9, 29–32], but since 2001, heroin-related overdose deaths have risen sixfold in the United States [33]. Heroin-related overdose intensified after 2010, with overdose mortality rates tripling between 2010 and 2014 from 1.0 to 3.4 per 100,000 [34]. The increase in heroin-related deaths has been paralleled by a rise in the death rate attributed to synthetic opioids other than methadone. The age-adjusted rate of overdose deaths attributed to synthetic opioids other than methadone, which includes fentanyl and its analogs, doubled between 2015 and 2016, rising to 6.2 per 100,000 [35]. Evidence from the US Drug Enforcement Agency indicates this increase is being primarily driven by illicitly manufactured fentanyl rather than diverted pharmaceutical fentanyl [36, 37]. While some have focused on the potency of fentanyl [38, 39] in increasing the risk for overdose, others have highlighted the risk of vicissitudes in the purity of fentanyl and its analogs in combination with heroin [40, 41].

In the face of overdose-related morbidity and mortality, numerous public health interventions have tried to address the risks of overdose over the last 20 years. These strategies include overdose education and peer naloxone distribution [42–44]. Promoting what has been termed “reverse transition” from injecting to a non-injecting route of administration constitutes another approach to reducing overdose risk [45].

One study of UK-based “chasers” and injectors found that while preferred administration routes were likely to persist for years and multiple route transitions were uncommon, 16% of their sample had reverse transitioned [46]. Another study of reverse transitions among heroin and cocaine users in New York City found that transition from injection to non-injection use appeared to be a relatively stable, long-term behavior change [47]. Among other transitions, several harm reduction organizations in the UK advocated for switching to rectal administration ("plugging"), the so-called “up your bum” campaign, but it is unclear how widespread this practice actually is [48, 49].

Given the low rate of “chasing” among US heroin users, one practice some injectors have adopted to reduce the risk of overdose is the use of “tester shots” or “test shots.” This entails injecting a small quantity of a drug sample in order to assess its potency qualitatively before deciding whether to inject the remainder of the dose [50–52]. A tester shot requires practitioners to perform two injections, a factor that may act as a barrier to the widespread adoption of this behavior, as venous access can become challenging for long-term injectors and higher injection frequency increases the risk of injection-related complications including viral transmission [2, 53].

Circumventing the multiple injection problem of the tester shot, “slow shots” allow the injector to insert the needle, release the tourniquet, and very slowly inject while assessing the embodied effect with the needle still in the vein [54]. A further refinement of this is the “graduated” or “controlled” shot where the needle remains in the vein, but the dose is divided into three with a pause for assessment of its effects between each third [55]. The term “tester shot” is used in this paper to encompass all forms of partial dose injection, while “drug sampling” refers to any method of administration involving a partial dose.

There is a dearth of qualitative research on behavioral adaptions that current heroin injectors are making with respect to the ongoing fentanyl adulteration crisis in the US. In this paper, we present findings from ethnographic fieldwork trips in 2015 and 2016 to Baltimore, Maryland; Worcester, Lowell, and Lawrence, Massachusetts; Nashua, New Hampshire; San Francisco, California; and Chicago, Illinois on embodied methods of gauging opioid strength that injection drug users in these areas are taking to prevent overdose. With the exception of California, where solid black tar heroin dominates, all these states have powder sourced from Mexico or Colombia and are suffering rising heroin- and fentanyl-related deaths.

In 2016, Baltimore lost 454 people to heroin-related overdoses, up from 260 the previous year, and 419 people to fentanyl-related overdoses, up from 120 in 2015 [56, 57]. The figures available for Massachusetts do not distinguish between heroin and prescription opioids. In 2016, among the 1374 individuals whose deaths were opioid-related (including heroin) and a toxicology screen was also available, 1031 of them (75%) had a positive screen result for fentanyl, an increase from 754 (57%) in 2015, although this may depend on the frequency of toxicological screening [58, 59]. Drug overdose deaths in New Hampshire increased by 1629% between 2010 and 2015, largely as a result of fentanyl. Hillsborough County, the location of Nashua, where 43.6% of the fentanyl deaths occurred, was most affected by these overdose deaths. The rate of death caused by fentanyl, heroin, and other opioids rose sharply between 2015 and 2016 [60]. In Chicago in 2016, there were 487 overdose deaths involving heroin and 420 involving fentanyl, both rising from the previous year [61]. In San Francisco in 2016, 41 deaths were attributed to heroin overdose and 22 attributed to fentanyl, doubling from the previous year [62, 63]. Data for 2017 are not available for all sites, but Baltimore showed a small decline in heroin-related deaths (from 334 in January to September 2016 to 305 in the same period of 2017) but a much larger increase in fentanyl-related deaths (from 276 January to September 2016 to 427 in the same period in 2017) [64]. Massachusetts experienced a modest decline in overall opioid deaths in 2017 but an increase in the proportion screening positive for fentanyl (to 83%) [65].

## Methods

The “Heroin in Transition” (HIT) study is conducting “hotspot” research where ethnographers are dispatched to locations around the country upon receiving reports of unusual or dangerous heroin or high levels of overdose [40]. HIT’s approach is adapted from “rapid assessment,” a short-term multidisciplinary research model aimed at gaining an insider perspective of social, economic or health problems and their possible solutions [66]. Originally developed in the 1970s, rapid assessment has enabled researchers to gain knowledge about emerging health problems in a short time period and has been used effectively in investigating health concerns related to drug use [67–69].

HIT uses highly focused ethnography to investigate new and evolving heroin forms and users’ responses, their methods and contexts of use [17]. The paucity of data regarding the risks associated with novel heroin forms of unknown purity and modes of use, along with contamination of the heroin supply with fentanyl [41] and the urgent need for knowledge acquisition to develop interventions, makes rapid ethnography highly appropriate to this time sensitive problem.

Unlike most rapid assessment, the HIT model is chiefly qualitative, with quantitative questions explored by the Heroin in Transition study outside the rapid assessment to inform the overall picture. Our research model aims to represent “from below” the experiences and perceptions of users in the current opioid epidemic. These data are placed alongside observations by the ethnographers based on their own experience of observing heroin and its use in other locations. Data are not formally triangulated, but we hope that a composite picture emerges from the multiple perspectives of users and ethnographers. Further, rapid ethnography allows us to generate hypotheses to be tested by quantitative research methods. Between 2015 and 2016, the ethnographic team visited Baltimore, Maryland (November 2015 and March 2016); San Francisco, California (February 2016); the Massachusetts cities of Lawrence, Lowell, Worcester and Nashua, New Hampshire (June 2016); and Chicago, Illinois (September 2016).

### Recruitment and data collection

Following information about fentanyl-laced and fentanyl-substituted heroin in Baltimore, New Hampshire and Massachusetts and high levels of overdose in Chicago, contact was made with harm reduction service providers in these locations. In San Francisco, the ethnographic team used personal contacts to arrange interviews with users and also carried out recruitment on the street. The study protocol was also approved by the University of California, San Francisco Institutional Review Board. The data and its collection are protected by a US Federal Certificate of Confidentiality issued by the National Institutes of Health/National Institute on Drug Abuse.

Data were collected through semi-structured interviews, ethnographic observation captured in collaborative field notes and video recording of heroin preparation and consumption. With its observation in the field and long established “ground-up” approach to data collection, ethnography is a method well suited for examining the experiences and phenomena of everyday life—and has been used widely in research among drug users [3, 70]. Rapid ethnography uses a more directed and condensed approach than traditional ethnography, collecting data with a particular set of questions in mind.

To be eligible for the study, participants had to be at least 18 years of age and self-reported current injectors whose primary drug was heroin. Exclusion criteria covered individuals who were intoxicated or otherwise unable to give informed consent or answer questions reliably. Researchers approached users attending needle and syringe program sites, explained the study to them, and obtained their consent. Some snowball sampling was also conducted [71]. Only verbal consent is sought to avoid the collection of participants’ full names or signatures. Pamphlets approved by UCSF IRB explaining the study and respondents’ rights were also provided. Interviews are conducted in as private a setting as possible. Before the interview begins, the research participants are reminded that they are free to decline to answer any question or to leave or stop talking to the researcher at any time. The team carries mobile phones for emergencies. Respondents received a small cash sum in compensation for their interview (approximately 0.5–1 h), aiming to balance respect for participants’ time while not acting as an inducement for users to participate [72]. All participants were interviewed once while some provided neighborhood tours or permitted the ethnographers to observe and film heroin injections.

The semi-structured interviews were carried out by the ethnographers (JO, EW, FMC and MH), project director (SM), and PI (DC) immediately upon recruitment at the needle and syringe program locations, in rental cars, cafés, users’ homes or drug-using locations. An interview guide provided a general structure to the conversation, including questions on the respondents’ life course of drug use, a typical day in their life involving drug acquisition and use, perceived changes in the heroin supply, knowledge of fentanyl, preferences for heroin vs. fentanyl, methods of use, perceived physiological effects and experiences of overdose.

After our initial visit to Baltimore indicated some tester shot usage among the population, focused questioning on drug sampling was incorporated into the interview guide for subsequent sites. All interviews were audio-recorded and transcribed in their totality and verified (by JO) against the audio recording. Field notes were drafted collaboratively each research day and finalized after the trips. The PI (DC) is a physician and can address participants’ medical concerns or questions or that arise during the flow of this research. As data is analyzed, findings are fed back to local service providers to assist in the development of tailored harm reduction responses.

### Sample characteristics

This was a non-random convenience sample. Across all sites, 91 current heroin injectors were interviewed (Baltimore, *n* = 22; Chicago, *n* = 24; Massachusetts and New Hampshire, *n* = 36; San Francisco, *n* = 9), of whom 62 were male and 29 were female. The experience injecting heroin ranged from less than a year to 47 years. Eight participants, who were dedicated heroin snorters, were also interviewed. Names have been changed to protect confidentiality.

### Analysis

Transcripts were read in their entirety, and text relating to tester shots/snorting and other harm reduction methods were extracted by JO and discussed by JO, DC, and SM, who then clarified categories of activity, motivation, provenance and other themes arising from the data. Observations from the field notes and video recording were also incorporated in the analysis. The analysis gave priority to the ways in which people experience heroin but also included the reflections of the ethnographers observing the drugs and their administration.

Some degree of subjectivity and contextual influence are present in all data interpretation, but the ethnographers maintained an awareness of this, discussing and examining their own positionality and preexisting ideas at multiple points during the research process. Data analysis was conducted by three multidisciplinary researchers with diverse life experiences, disciplinary backgrounds, age, and mixed genders. Where discrepancies in the interpretation of the findings arose, these were discussed until agreement was reached.

## Results

### Motives

The unpredictability of heroin potency is a long-established problem of its illicit supply, particularly for injectors whose dose is delivered in a single “bolus,” putting them at increased risk of overdose relative to those who insufflate (toot, snort or sniff) or smoke heroin. Users become aware of these risks in a number of ways, including personally experiencing or witnessing overdoses or losing friends or partners. The research study’s first encounter with drug sampling was with a young man in San Francisco who, after experiencing an overdose, had started smoking the strong black tar heroin known locally as “point dope” before judging how much to inject:

I’ll smoke it just to see how I feel from taking a couple of hits and then I’ll only do a point [0.1g] or if I’m really spooked, like, even less, just try it out. […] Yeah, you know what’s funny though, I didn’t always do that until I had OD’ed and then I was like “Oh fuck! Now I’m playing with my life here.” (Riley, in his 20s, using for 7 years)

Like Riley, some interviewees mentioned adopting drug sampling as a result of experiencing overdose firsthand, but others did so after observing others. Liz from Lawrence, MA, a woman in her 20s who had been injecting for 1 year and had never personally overdosed, explained that she had adapted her behavior with fentanyl-laced heroin, “Because you learn from everybody else being stupid”:

[…]when I get stuff I don’t know what it is, I do a little bit before I do something that I feel. Like I want to kind of scale out how much I want to do. Because I don’t want to die. But these people are just doing a gram shot and just… my friend just died two days ago.

While some referred to these methods as their regular practice, others sampled in particular circumstances such as after periods of abstinence or when the heroin or its source were unfamiliar. In Chicago, David, who was in his 30s and had been using for 7 years, generally relied upon a regular dealer to regulate the quality and strength of his heroin, as well as keeping his doses small, but he snorted heroin prior to injecting when buying from an unfamiliar source:

Q: Do you ever taste a new bag?

A: No, because I’ve been going through the same guy and it’s just always consistent. His is always, you know, straightforward. You know what you’re going to get. So I trust it. But if I were to go to somebody else that I hadn’t gone to before I would definitely taste it, snort a little bit, or you know if I were to shoot it, I would shoot a small amount. Because you can always shoot more, you can never shoot less.

### Drug sampling methods

Users described a wide variety of methods for drug sampling, sometimes creatively combining them together, from using non-injecting routes of administration such as snorting, smoking or tasting a small amount prior to injection to injecting a partial dose and waiting. Partial injection could take different forms: either a “slow shot” where the user injected a portion of the solution in the syringe, keeping the needle in the injection site, and then either continuing or withdrawing the syringe or a “tester shot” where the solution was divided between two or more separate injections. Other techniques included getting feedback from other users who were using heroin of the same batch or observing other users with higher tolerance injecting heroin from the same batch before judging how much to inject themselves.

#### Drug sampling by snorting and tasting

In Chicago, where the powder heroin can be snorted rather than smoked, several injectors described snorting their heroin before injecting. Ray, in his 50s and using for 25 years, explained that this not only gave an indication of its strength but also a taste at the back of the throat which, he believed, was indicative of its ingredients:

A: […] I’m not the only one that do this. There’s a lot of my buddies that do heroin too, will toot [snort] it first before we even start cooking it up to see what’s it about. […]See, when you toot dope it’s supposed to go down your nose and it’s supposed to go down smooth without no burn or none of that… Now, once you get that drain, it’s the taste. It tastes like dope. Because see, some dope that tastes like aspirin ain’t dope…. So you get familiar with the taste of dope too once you do it, once you had done it so long with me…. So once you get all that in perspective, […] the taste, the smoothness going down, the drain, you’ll start feeling a nice, warm sensation. So now I know it’s straight [heroin] so it’s time for me to party now.

#### Tester shot

Several of our research participants indicated that they had begun injecting a smaller amount of heroin to assess qualitatively its embodied effects before injecting a larger dose. Johnny in Chicago, in his 20s and using for 6 years, was among those who endorsed this method:

A: […] I’m very cautious on my heroin intake. I like to know how much I’m doing. And if anything, I always do a little bit less and sometimes I get pissed off because I don’t get high from the little shot.

Q: So you take care of yourself, testing a little bit.

A: I always test the waters before. You don’t just jump in with heroin.

Glen, in his 20s, using for approximately 1 year in Baltimore, described his method of tester shot:

Q: How long are you waiting to judge how strong it is?

A: I’d say anywhere from 5 to 15 minutes.

Q: 5 to 15 minutes. You’re a patient guy. I imagine a lot of other people aren’t so patient.

A; Well, it’s… pays off in the end, I guess.

#### Half-dose slow shot

Some of those interviewed had managed to avoid overdose entirely, perhaps as a result of using drug sampling methods. Larry, a Baltimore man in his 60s who had survived over 40 years of heroin use without overdosing, explained how he injected half his shot of heroin, waited a short time, and then decided whether to inject the rest. If he decided to inject the rest of the shot after waiting, he registered to check whether he was still accessing the vein first. We asked him about how he managed the decision not to use the whole shot:

Q: So what happens if you’re feeling like that half shot is really good, do you have enough self-control to take the rest of it out and not do it?

A: Yeah, I can. And I also like to have it where I can put the rest of it in me, because I feel as though my intake can allow me to take the rest of that there.

#### Combined tester and slow shots

James, a Baltimore man in his 50s who had been injecting for half his life and never overdosed, had recently experienced a number of friends dying from overdoses. His method involved loading the syringe with half the dose and then further dividing the half doses for a slow shot:

A: […] I don’t, what they call, slam it and put the whole thing in me. I do it little by little just to see what the effects is and stuff.

Q: So do you like have one syringe and you use a little bit of it or do you like have several syringes?

A: No, just one syringe and one syringe I put what we say 80 on the height. So once I cook it up and do what I’m going to do with it, and most of the time I’ll speedball, you know, cocaine and heroin. And I will take 40 of it and I’ll take the other 40 and put it to the side and then when I hit myself I’ll just put 20 in it just to see, you know, what the effects is …

#### Combined user report and slow shot

Janice, also a long-term user in Baltimore, in her 50s, who had also never overdosed, used a combination of approaches to regulate her intake. This involved getting an oral report from a friend, gauging how much to dilute the heroin depending on her friend’s reaction, and then taking a half-dose slow shot, keeping the needle in place before deciding whether to use more.

Q: … Since you don’t know whether it’s going to be weak or strong how do you manage that?

A: What I do is I get a reading from someone else because I know myself.

Q: A reading?

A: Meaning they tell me how it is for them. And that determines how much water I’ll put on mine. Like my girlfriend might say, 'Girl, that dope is good as a motherfucker.' I might put 40 on it and I’ll put a 60. […]

Q: You dilute it if the strength is good?

A: Yes.

Q: All right. Then how do you inject that 60? […]

A: 30.

Q: You’ll do 30?

A: I’ll do 30, wait for it to hit me and see if I can handle it or do I need to come out.

Q: How long you waiting for?

A: As soon as it hit me. Which usually takes I’d say 20 seconds.

Q: 20 seconds, okay. And then you just decide to pull out or --?

A: Or go ahead with it.

#### Mixed heroin-tolerance partners

Relying on friends’ experiences of a single batch was also something we observed in Baltimore. Glen and Scott are running buddies sharing resources including heroin [3]. Glen had only started injecting in the last year after a brief period snorting heroin following his transition from opioid pills. Scott, in his 40s, had a higher opiate tolerance after using heroin for many years and acted as “taster.” In this case, Glen heated a speedball in a single cooker, dividing the solution equally between two syringes. After Glen had injected Scott in his neck, Scott reported on its strength and advised Glen to go ahead and inject his own dose. Although Glen’s tolerance was lower, they shared equal doses, perhaps an equity demanded by their friendship, but took care to keep each other safe.

### Users’ reflections on drug sampling

However, there are limits to the drug sampling strategies just described, especially in the age of fentanyl. Harry, a user in his 40s from Lowell, MA, using for 20 years, explained that even with a tester shot, he had almost overdosed from heroin he later believed to contain fentanyl:

I almost overdosed, I almost died. I almost went out. And I didn’t do much, it was kind of like a tester shot and it was really powerful. I just thought it was really good dope, you know? I didn’t know it was fentanyl until it started getting around that the fentanyl was floating around…

Whether users developed these methods themselves, learnt them from peers, publicity campaigns or harm reduction workers was often unclear. However, an awareness of the irreversibility of injection was a recurring theme.

I’m taking my time because if I’m gonna mix it with 65 [units of water], I’m gonna put 10 or 15 in me to see the quality of what I’m putting in me, because you can always put more in, but can’t take it out. I’m a firm believer in that.—Doug, Baltimore, in his 40s, using for 32 years

I’ll do a little bit at a time just to make sure I’m not – that I’m cool. And then you can always give yourself more but you can’t take back.—Joanie, Chicago, in her 30s, using for 14 years

Q: Do you ever do a tester shot or do you use the whole thing in one shot?

A: It depends on the drug, sometimes I don’t do it all at once. You have to respect dope; if you know it’s strong, you have to use it carefully.—Gonzalo, Nashua, NH, in his 50s, using for 20 years

Q: And you were saying a bit about how you avoid ODing yourself?

A: Oh yeah, because I don’t, you know, push the whole thing in me at one time, because like I said, there is no pulling it back […] once you push it in you, ain’t no trying to pull it back […]—James, Baltimore

The presence of overdose as an imminent danger was especially pervasive among users in Baltimore and Massachusetts, many of whom had lost friends, relatives or acquaintances. Although a minority of those interviewed described using these drug sampling techniques, there is clearly receptivity among some users to protecting themselves by using a variety of methods.

## Discussion

Our ethnographic research shows that in the five states visited for this study, some people using heroin have incorporated tester shots and other drug sampling strategies into their injection practices. These included a wide range of approaches, sometimes in combination, in which people used their own or others’ embodied experiences to judge the strength of the drug qualitatively before deciding on their dose. While these methods do not guarantee safety from an overdose, particularly in the cases of some of the more powerful fentanyls, it would be valuable to test epidemiologically whether those using drug sampling methods report experiencing fewer overdoses.

Peer advocacy for tester shots and drug sampling may be the most effective means of expanding the use of these methods. A number of research studies conducted in the US and internationally have demonstrated the connection between social norms, peer adoption, self-efficacy and perceived acceptability in the successful adoption of harm reduction strategies [73–77]. Other research has demonstrated how social norms within drug-using groups influence the perception of risks and acceptable practices [78]. The promotion of drug sampling methods between peers as a form of harm reduction therefore needs to be done in a way that reflects the structural risks of the regional drug economy and an awareness of local norms among users. For instance, to advise users to sample their heroin by smoking before injecting might be suitable where a base form of the drug, such as Afghanistan-sourced heroin, is dominant, but in the case of US powder heroin, much of the drug would be lost before absorption and snorting prior to injection would be a better option. Alternatively, where heroin prices are high and/or availability is low, tester shots may be more appealing than snorting. Aside from these market considerations, further research into facilitators and barriers to the uptake of drug sampling is needed. Barriers suggested to date are a loss of intensity from spreading the dose over time and “wasting” heroin through less effective delivery methods such as snorting [26, 79].

Psychologically informed approaches may also shed some light on how best to encourage the use of drug sampling. One survey of the self-efficacy of harm reduction practices, including tester shots, found that adherence to all harm reduction strategies surveyed was markedly lower when respondents were asked to imagine being in withdrawal [80]. A later study identified being in a hurry to use (24%) and purchasing drugs from a known dealer (20%) as the most significant barriers to the adoption of tester shots [81]. Health beliefs about perceived susceptibility to and severity of overdose may influence the adoption of overdose-related harm reduction methods such as tester shots [82]. Many of those we spoke with reported the recent deaths of friends, family and acquaintances [17, 41], as well as experiencing their own overdoses. We do not know whether decisions to use drug sampling strategies are shaped more by psychological or situational factors.

In order for tester shots and other drug sampling strategies to be effective at preventing overdose, people who inject heroin must be willing to use them consistently, since risk is difficult to determine at the point of use. Other interventions that may be effective aids in assessing overdose risk include point-of-use testing, also known as drug checking, and qualitative user discernment of street heroin. Recent research has shown a high willingness among younger, US-based injection drug users to utilize fentanyl test strips [83], but the hypersensitivity of on-the-market fentanyl test strips raises questions about their efficacy in helping injectors to quantify risk. Harm reduction interventions focusing on “universal precautions,” i.e., consistent use of drug sampling, may be more effective than point-of-use fentanyl testing. This and the utility of combined interventions need to be evaluated. User discernment of fentanyl-adulterated and -substituted heroin may also be a useful supplement to both drug sampling and point of use testing [40].

This study is explorative qualitative research based on a convenience sample of heroin injectors in five US states. Due to the non-random sampling methods and semi-structured interview format, quantitative comparisons of drug sampling patterns across the research locations or by sample characteristics should not be made. The data comprise a snapshot of drug consumption behavior that can generate hypotheses but not conclusive findings. Interviews carried out at needle and syringe programs and other public health settings may be influenced by social desirability bias. Some interviewees were recruited outside of these environments, however, in an effort to mitigate this bias.

## Conclusions

Harm reduction services are central in the promotion of safer drug use techniques. Bearing in mind the non-random study sample, we found heroin sampling methods to be most common in Baltimore and Chicago, where robust harm reduction services have been established for more than two decades (Baltimore’s in 1994 and Chicago’s in 1991). In Massachusetts, two of the three cities visited had no official needle and syringe programs, while Worcester’s had only started in 2016, and few users mentioned using tester shots or other drug sampling methods. In Chicago, where most of those using tester shots and snorting were interviewed, the local harm reduction service, Chicago Recovery Alliance [84], has been teaching these methods for two decades. Snorting, for those willing to do so, was considered a particularly informative method for evaluating the drug as it included other sensations, allowing users to detect pH balance, additives, and other drug characteristics.

The use of tester shots and other drug sampling methods as a means of preventing an overdose from injection drug use reduces the quantity absorbed at any one time, allowing users to monitor for drug strength and titrate their dose accordingly. In the face of greater perceived susceptibility to and severity of opioid-related overdose due to the rising presence of fentanyl-adulterated and -substituted heroin [40], this could be a useful strategy to reduce both morbidity and mortality.

## Abbreviations

HCL: Hydrochloride
HIT: Heroin in Transition
US: United States
